# The benefit of a geriatric nurse practitioner in a multidisciplinary diagnostic service for people with cognitive disorders

**DOI:** 10.1186/s13104-015-1189-6

**Published:** 2015-06-04

**Authors:** Bart H L Ament, Claire A G Wolfs, Gertrudis I J M Kempen, Ton Ambergen, Frans R J Verhey, Marjolein E De Vugt

**Affiliations:** CAPHRI School for Public Health and Primary Care, Maastricht University, PO Box 616, 6200 MD Maastricht, The Netherlands; Alzheimer Centre Limburg, MHeNS School for Mental Health and NeuroScience, Maastricht University, PO Box 616, 6200 MD Maastricht, The Netherlands; Department of Health Services Research, Maastricht University, PO Box 616, 6200 MD Maastricht, The Netherlands

**Keywords:** Dementia, Integrated care, GP concordance, Geriatric nurse practitioner

## Abstract

**Background:**

The aim of the study was to evaluate whether adding a geriatric nurse practitioner (GNP) to an outpatient diagnostic multidisciplinary facility for patients with cognitive disorders (Diagnostic Observation Center for PsychoGeriatry, DOC-PG) could improve quality of care. DOC-PG combines hospital diagnostics and care assessment from a community mental health team and provides the general practitioner (GP) with advice for treatment and management. In a previous study, we found that 28.7% of the advice made by this service was not followed up on by the GP.

**Methods:**

Two cohorts were studied: a group of patients with added GNP (n = 114) and a historical reference sample (n = 137). Both groups followed the same diagnostic protocol and care approach, but, in the GNP group, a care coordinator was added in order to communicate the advice from the DOC-PG to the GP. The primary outcome was the concordance rate of GPs regarding the advice. At the patient level, health-related quality of life (HRQoL) was assessed. Self-Rated Burden and care-related quality of life were measured at the informal caregiver level. Measures were conducted immediately after DOC-PG diagnosis and after 6 and 12 months. Univariate analyses, logistic regression analyses, and mixed model multilevel analyses were used to test differences between both groups.

**Results:**

Total concordance rates were significantly higher in the GNP group compared to the reference sample (82.1 and 71.3%, respectively; *p* < 0.001). No improvement in patient HRQoL was identified. Among the informal caregivers, a significant reduction of Self-Rated Burden was found in the GNP group at 12 months (adjusted mean difference −1.724, 95% CI −2.582 to −0.866; *p* < 0.001).

**Conclusions:**

Adding a GNP to an outpatient diagnostic multidisciplinary facility for patients with cognitive disorders may improve the GP concordance rate of the advice from the DOC-PG and reduce subjective burden of the informal caregiver.

## Background

Timely recognition and accurate diagnosis of cognitive disorders, such as dementia, are crucial for improving care for both patients and their informal caregivers [1]. Specialized services, such as multidisciplinary memory clinics, can facilitate an early diagnosis by providing the referrer with thorough physical, neuropsychological, functional, and psychiatric assessments leading to specific advice or recommendations. The effects of an integrated multidisciplinary approach to dementia have been investigated in several studies [2–5]. Results suggest that such an approach is cost-effective and has a positive impact on the quality of life of patients and their informal caregivers [5].

Early diagnostics can, however, only be effective if they are translated into advice and a treatment and care plan that is followed up on by the referrer. The extent to which recommendations are followed up on by a referrer has been studied in various patient groups [6–10]. Compliance with recommendations for geriatric patients is generally reasonable (69–77%), and is dependent on the type of advice [7, 11]. Wolfs and colleagues [11] found that general practitioners (GPs) are likely to implement the recommendations made by an outpatient diagnostic multidisciplinary facility for psychogeriatric patients. However, they also concluded that recommendations with respect to referral to physical therapists and occupational therapists showed a rather low rate of concordance. In addition, the role of both disciplines in managing psychogeriatric patients might not be well known. Noncompliance is often related to failures in communication, not only between the GP and patient, but also between generalist and specialist services [7, 12]. Improved coordination of advice and recommendations could lead to an increase in concordance. We now present a study in which the additional value of a geriatric nurse practitioner (GNP) to an outpatient diagnostic multidisciplinary facility for psychogeriatric patients is examined, in order to improve the quality of care. The Diagnostic Observation Center for PsychoGeriatry (DOC-PG) is an example of such an outpatient diagnostic multidisciplinary facility for psychogeriatric patients. The GNP is appointed to improve the communication between GP and DOC-PG, and to coordinate and monitor advice. We hypothesized that adding a GNP who acts as a linking-pin between the GP and the outpatient diagnostic facility would have beneficial effects on three levels, i.e., (1) GP level, (2) patient level, and (3) informal caregiver level. At the GP level (1), effects were expected relating to compliance of advice. Advice given by an outpatient diagnostic facility to the GP was expected to be followed more closely if mediated by a GNP. At the patient level (2), a higher health-related quality of life (HRQoL) was expected due to higher concordance rates of advice. At the informal caregiver level (3), subjective care burden was expected to be lower due to higher concordance rates of advice, and care-related quality of life (CarerQoL) was expected to be higher.

## Methods

### Design and sample

**Figure 1:**
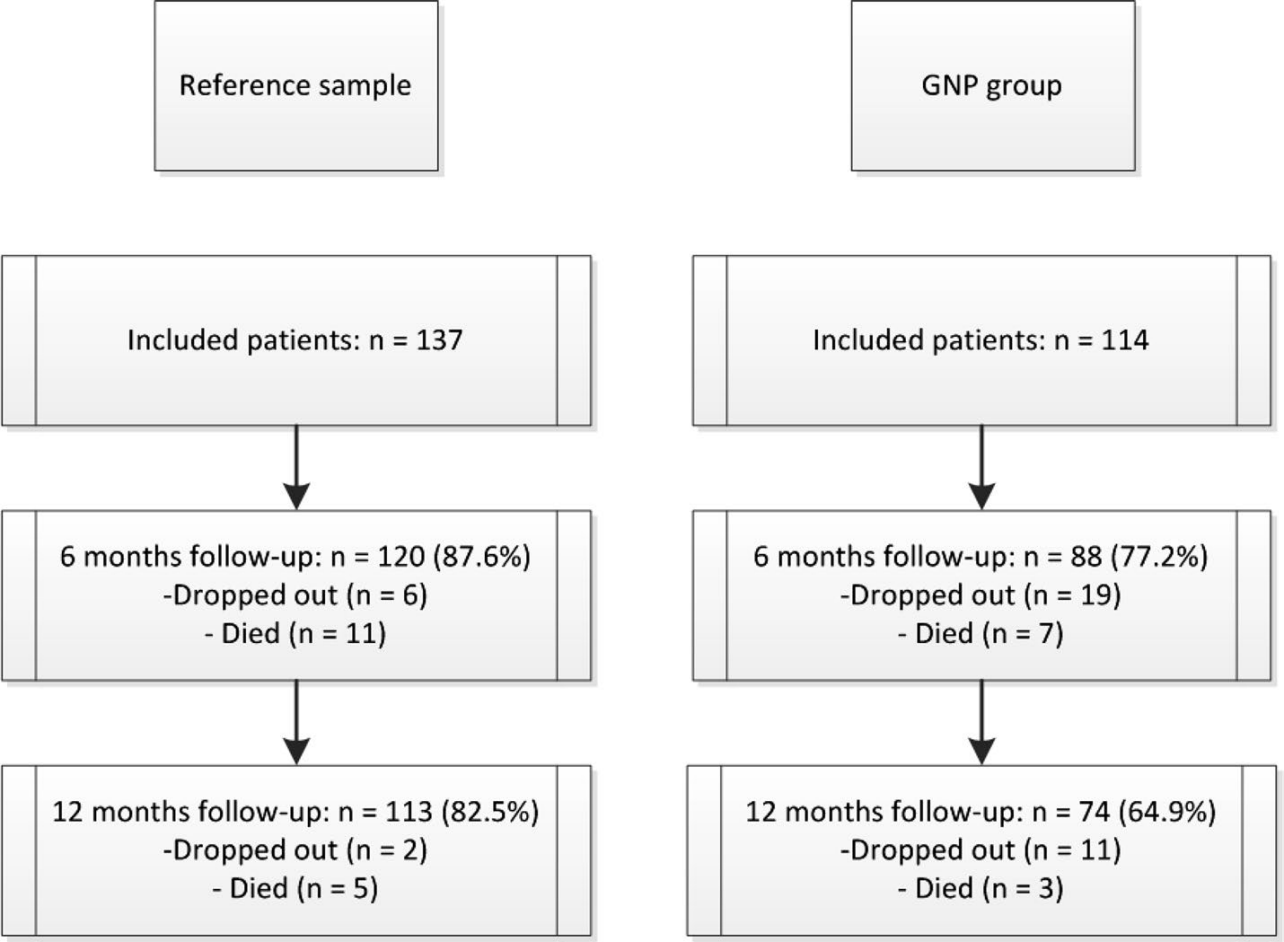
Flow chart study design and participants.

An observational study design with two (sub)cohorts was used: a cohort of participants where a GNP was added and a historical reference sample (see Figure 1 for a description of the number of participants at each stage of the study). The reference sample was recruited between July 2002 and August 2004, and was part of the MEDICIE study, which evaluated the effects of an integrated and multidisciplinary approach for psychogeriatric patients (the DOC-PG). The GNP group consecutively enrolled in the study between March 2010 and September 2011, and followed the same multidisciplinary procedure as the reference sample with the exception that, in the GNP group, a GNP was added to the DOC-PG team. Based on a power calculation, which ensures the detection of at least 80% of the differences in the mean score on the visual analogue scale (VAS) of the EQ-5D at 5% significance, 116 patients per group needed to be included at baseline. The Medical Ethics Committee of the Maastricht University Hospital Medical Center approved the study.

### DOC-PG team including GNP

The DOC-PG is a facility that provides multidisciplinary assessment and therapeutic advice for patients with cognitive disorders. Detailed information about the DOC-PG can be found elsewhere [5]. One GNP has been appointed within the DOC-PG team to communicate the advice from the DOC-PG to all the GPs who referred patients to the DOC-PG. After screening and assessment of the patient by the DOC-PG team, the results of the diagnostic process are discussed with the patient and his or her informal caregiver by the geriatric psychiatrist and the GNP (this discussion also took place in the reference sample with the exception that no GNP was present). Immediately after this disclosure, the GNP contacted the GP by telephone and discussed the diagnosis and advice given by the DOC-PG team; together, they allocated the tasks and formulated a plan of action. The GNP monitored this plan of action and registered the care-trajectory in consultation with the GP. In addition, the informal caregiver could contact the GNP whenever necessary. In summation, the GNP functioned as a mediator between the DOC-PG facility and the GP and, in addition, was a contact person for informal caregivers and patients. In the reference sample, the GPs only received a summary of the assessments, diagnosis and recommendations for treatment and management by written communication.

### Measures

#### GP level

For both cohorts, compliance with advice formulated by the DOC-PG team was checked by means of a concordance checklist, which was sent to each GP who referred a patient to the DOC-PG during the inclusion period. The concordance checklist listed all advice or recommendations from the multidisciplinary team at the patient level from July 2002 to August 2004 (reference sample), and from March 2010 to September 2011 (GNP group). The GP was requested to indicate whether advice from the DOC-PG team had been followed or not (yes/no). Advice was also scored as concordant if it had already been carried out (i.e., patients already received the care that had been recommended) or in case a patient refused to comply with a recommendation for treatment. GPs who did not respond received multiple reminders (either by phone or by mail).

#### Patient and informal caregiver level

To assess the outcome measures on the patient and informal caregiver levels in the GNP group, a self-administered questionnaire was used, which was filled out by the informal caregiver (i.e., rated by proxy). The questionnaire consisted of the following measures. HRQoL of the patient, which was measured using the EQ-5D [13]. This instrument provides a simple descriptive profile (utility score) and a single index value for health status (i.e., a VAS). The EQ-5D was used because patients were expected to show a complex range of mental, physical, and social problems. In both cases, higher scores indicate better health states. The subjective burden of the informal caregiver was measured using the Self-Rated Burden scale (i.e., VAS score with higher scores indicating a heavier burden) [14]. CarerQoL was measured using the CarerQoL (VAS score with higher scores indicating a better quality of life of the informal caregiver) and the CarerQoL-7D [15], which measures seven dimensions of the burden of caregiving. A weighted sum-score was used ranging from 0 to 100, where 0 is the worst caregiving situation and 100 is the best caregiving situation. The first questionnaire (T0) was given to the informal caregiver when the DOC-PG facility was visited for the diagnostic assessment. A second and third questionnaire were sent to the informal caregiver by mail 6 months (T1) and 1 year (T2) after visiting the DOC-PG facility.

### Statistical analyses

Background characteristics of patients and informal caregivers were summarized using descriptive statistics. Subsequently, the GNP group and the reference sample were compared regarding these characteristics by means of t-tests for continuous variables and *χ*^2^ tests for categorical variables.

For the analyses regarding concordance rates, both cohorts were compared using *χ*^2^ tests. Additionally, a logistic regression analysis was used to examine the influence of the covariates that follow: type of practice (group or solo), age, sex, living situation of the patient (alone or not alone), and the mini mental state examination (MMSE) score on concordance of the advice that was associated in a univariate way. We expected that these covariates, in particular, could influence the concordance rate of the advice.

With respect to outcomes on the level of patients and informal caregivers, mixed model multilevel analyses were used to examine differences between groups, i.e., the influence of the GNP as the main predictor of interest on outcomes. Cohort was considered the independent variable. Baseline scores of the dependent variable were included in the model as the covariate. Additionally, age, gender, living alone or not, and MMSE score at baseline were included in each model as a covariate. GP was added as an extra level in the model since patients were referred to the DOC-PG by different GPs. Missing values for the EQ-5D and the CarerQoL-7D were imputed at the scale level by means of multiple imputations based on the guidelines of the developers. Multilevel analyses are robust against missing values at the measurement level. At least the baseline measurement and one out of two follow-up measurements was needed to be included in the analyses. This explains the variation in the number of cases per outcome measure and why numbers differed between those mentioned in the results of the analyses and the flow chart. The software used for the analyses was SPSS version 20.

## Results

Baseline characteristics of the patients and informal caregivers are summarized in Table 1. Cohorts significantly differed regarding age of the patient and experienced burden of the informal caregiver.

**Table 1:** Baseline characteristics for patients and informal caregivers

	Reference sample	GNP group
Patients	N = 137	N = 114
Age^a^	78.3 (6.53)	81.9 (5.75)
Women	89 (63.1%)	70 (61.4%)
Diagnosis		
Dementia	97 (70.8%)	84 (73.7%)
No Dementia	40 (29.2%)	30 (26.3%)
MMSE	20.39 (5.61)	20.48 (4.01)
Informal caregivers		
Age	60.12 (13.77)	62.53 (12.30)
Women	90 (65.7%)	68 (59.6%)
Outcome measures		
EQ5D utility score	0.579 (0.289)	0.523 (0.289)
EQ5D VAS	47.91 (20.91)	51.67 (17.66)
Self-Rated Burden^b^	3.42 (2.81)	4.54 (2.72)
CarerQoL VAS	7.18 (1.71)	6.94 (1.44)
CarerQoL 7D score	82.10 (15.34)	77.39 (17.83)

Data are mean (SD) or number (%), GNP group = cohort with geriatric nurse practitioner.

p = ^a^<0.001, ^b^ 0.004.

### GP level

The results concerning concordance rates are summarized in Table 2. Respectively, 89.9 and 83.3% of the GPs returned the concordance checklist for the reference sample (44 of the 49 GPs) and the GNP group (50 of the 60 GPs). Total concordance rates differed significantly between the two cohorts (*p* < 0.001), with 71.3 and 82.1% for the cohort without and with the GNP, respectively. Regarding types of advice, significant differences were found for the categories ‘adapt medication’ (*p* = 0.014), ‘refer to hospital’ (*p* < 0.001), and ‘refer to healthcare professionals’ (i.e., physical therapist, occupational therapist, dietitian, speech and language pathologist) (*p* = 0.002). The DOC-PG cohort with GNP scored higher on concordance rates in these four categories.

**Table 2:** Concordance rates for reference sample and GNP group

Type of advice	Reference sample	GNP group
	Concordance rate	Concordance rate
Adapt medication^a^	73/88: 83.0%	96/102: 94.1%
Perform diagnostics	65/84: 77.4%	39/51: 76.5%
Arrange follow-up	18/24: 75.0%	50/59: 84.7%
Give general advice	41/57: 71.9%	74/92: 80.4%
Provide psycho education	9/14: 64.3%	17/19: 89.5%
Arrange home care	27/32: 84.4%	16/20: 80%
Arrange admission to nursing home	14/15: 93.3%	7/7: 100%
Arrange admission to care home	2/2: 100%	13/13: 100%
Arrange daily activities (day care)	23/27: 85.2%	19/26: 73.1%
Refer to MC for AD medication	26/30: 86.7%	33/43: 76.7%
Refer to hospital (not DOC-PG)^b^	38/70: 54.3%	139/164: 84.8%
Refer to allied healthcare professionals^c^	42/87: 48.3%	78/112: 69.6%
Total^d^	378/530: 71.3%	581/708: 82.1%

Data are: concorded advice/total advice: percentage of concordance, GNP group = cohort with geriatric nurse practitioner.

p = ^a^0.014, ^b^ 0.000, ^c ^0.002, ^d^ 0.000.

The results of the logistic regression analyses are shown in Table 3. Concordance was higher for the GNP group (compared to the reference sample) with respect to ‘total advice’ (OR 1.81, 95% CI 1.37–2.40; *p* < 0.001), ‘adapt medication’ (OR 3.07, 95% CI 1.05–8.94; *p* = 0.040), ‘refer to hospital’ (OR 4.58, 95% CI 2.34–8.96; *p* < 0.001), and ‘refer to healthcare professionals’ (OR 2.51, 95% CI 1.35–4.68; *p* = 0.004).

**Table 3:** Logistic regression analyses regarding type of advice

Type of advice	OR	[95% CI]	*p*
Total			
Cohort	1.80	[1.37–2.37]	0.000
Practice (group (1)/solo (2))	0.57	[0.43–0.75]	0.000
Living alone (1)/not living alone (2)	0.92	[0.77–1.11]	0.403
MMSE	1.03	[0.99–1.05]	0.057
Medication			
Cohort	2.99	[1.05–8.53]	0.040
Practice (group (1)/solo (2))	0.25	[0.09–0.74]	0.012
Living alone (1)/not living alone (2)	0.90	[0.44–1.83]	0.760
MMSE	0.98	[0.88–1.08]	0.654
Refer to hospital			
Cohort	5.01	[2.60–9.65]	0.000
Practice (group (1)/solo (2))	0.70	[0.36–1.35]	0.283
Living alone (1)/not living alone (2)	0.97	[0.65–1.45]	0.877
MMSE	1.06	[1.01–1.13]	0.034
Healthcare professionals			
Cohort	2.38	[1.31–4.34]	0.005
Practice (group (1)/solo (2))	0.45	[0.24–0.82]	0.010
Living alone (1)/not living alone (2)	0.99	[0.68–1.48]	0.996
MMSE	1.01	[0.96–1.07]	0.685

The unadjusted scores for both groups and the adjusted mean differences between both groups for the primary outcome measures are summarized in Table 4.

**Table 4:** Mixed-effects multilevel analyses adjusted for baseline values and age, gender, living alone or not and MMSE score on baseline

	Reference sample	GNP group	Adjusted mixed-effects Mean difference [95% CI]
Patients			
EQ-5D utility score	N = 115	N = 71	
6-month follow-up	0.634 (0.270)	0.503 (0.275)	−0.092 [− 0.166 to −0.018]^a^
12-month follow-up	0.572 (0.296)	0.449 (0.274)	−0.092 [− 0.167 to −0.017]^b^
EQ-5D VAS score	N = 115	N = 72	
6-month follow-up	49.39 (17.62)	49.95 (17.34)	−0.387 [−5.472 to 4.697]
12-month follow-up	53.31 (18.27)	50.47 (17.92)	1.900 [−3.715 to 7.515]
Caregivers			
CarerQoL VAS	N = 100	N = 67	
6-month follow-up	7.12 (1.71)	6.76 (1.64)	−0.238 [−0.708 to 0.232]
12-month follow-up	7.00 (1.71)	7.09 (1.41)	0.103 [−0.371 to 0.577]
CarerQoL Tarif	N = 100	N = 100	
6-month follow-up	82.46 (14.52)	78.48 (14.06)	−3.116 [−7.149 to 0.916]
12-month follow-up	78.87 (16.04)	78.48 (15.13)	0.371 [−4.242 to 4.983]
Self-Rated Burden	N = 100	N = 83	
6-month follow-up	3.86 (2.56)	4.43 (2.54)	0.27 [−0.757 to 0.816]
12-month follow-up	4.09 (2.57)	2.80 (1.69)	−1.724 [−2.582 to −0.866]^c^

Data in columns reference sample and GNP groups and are unadjusted scores (SD). Numbers vary due to missing values. For the EQ-5D utility score, EQ-5D VAS score, and CarerQoL, higher scores indicate better functioning; for the Self-Rated Burden, lower scores indicate better functioning. GNP group = cohort with geriatric nurse practitioner.

p = ^a^0.015, ^b^ 0.016, ^c ^0.000.

### Patient level

No significant group by time interaction effects were found either for the EQ-5D utility score or for the EQ-5D VAS score. A group effect was found for the EQ-5D utility score. Patients in the GNP group scored significantly lower on the follow-up measurement after 6 months (adjusted mean difference −0.092, 95% CI −0.166 to −0.018; *p* < 0.05) and after 12 months (adjusted mean difference −0.092, 95% CI −0.167 to −0.017; *p* < 0.05) compared to the reference sample.

### Informal caregiver level

A significant group by time interaction effect was found for the Self-Rated Burden scale (*p* < 0.001). Scores on the Self-Rated Burden scale for the reference sample remained stable over time, whereas these scores for the GNP group decreased. This resulted in a significant difference between groups (adjusted mean difference −1.724, 95% CI −2.582 to −0.866; *p* ≤ 0.001) at the follow-up measurement after 12 months, indicating lower levels of burden in the GNP group. No significant differences were found regarding the CarerQoL-VAS and the CarerQoL-7D.

## Discussion

In general, the results of the study showed that adding a GNP significantly improved the concordance rate of GPs. Although the higher concordance rate did not lead to improved patient HRQoL, we found a significant improvement in the burden experienced by the informal caregiver.

The overall concordance rate is significantly higher in the GNP group compared to the reference sample. More importantly, recommendations that previously showed a particularly low concordance rate in the reference sample (i.e., referral to physical therapists and occupational therapists, psycho-education, referral to specialists in the hospital) were better adhered to in the GNP group. As a linking-pin between the GP and the DOC-PG, the GNP was able to mediate and to convince the GP of the importance of the advice or even carry out some of this advice from the GP. GPs rather left recommendations concerning other healthcare professionals (e.g., arranging physical and occupational therapy) to the GNP, and especially these recommendations were, to a lesser extent, complied with in the reference sample.

With respect to the experienced burden of the informal caregiver, a significant improvement was found for the GNP group after 12 months. How may a significant increase in the concordance rate for certain advice (i.e., referring to a hospital, referring to paramedical disciplines, adapting medication) account for a decrease in experienced burden? The tasks of the GNP in this study are partly comparable to those of a case manager. No consensus exists about how case management should be organized in the care for people with dementia. Nevertheless, care coordination helps informal caregivers to navigate through the healthcare system more easily and, in addition, reduce the fragmentation of care [16]. In this way, the GNP might decrease some of the many factors that influence informal caregiver burden such as feelings of helplessness, social isolation, and loss of autonomy. Those factors are found to be largely responsible for the experienced burden of informal caregivers of older people with dementia [17–20]. As mentioned earlier, informal caregivers were given the opportunity to contact the GNP with questions or problems regarding the care-trajectory. Even if they do not actually use this, it may give them some feeling of support [21]. In addition, advice, which may structure the daily life of patients and informal caregivers (i.e., physical therapy, occupational therapy, and psycho-education), were followed more closely in the GNP group than in the reference sample. This might have increased the informal caregiver’s sense of competence and, therefore, reduced the burden, as informal caregivers are involved in the process during the course of the disease. Effects of occupational therapy on improvement in patients’ daily functioning and associated informal caregiver burden have been reported elsewhere [22].

With respect to HRQoL on the patient level, results are mixed; no effect with respect to the EQ-5D VAS scores could be identified. There is, however, a significant group effect for the EQ-5D utility scores indicating that the GNP group scored significantly lower after 6 and 12 months. Patients in the GNP group were significantly older compared to the patients in the reference sample. It is hard to achieve any improvement in domains like HRQoL in dementia because of the progressive nature of the disease. Because of this progressive nature of the disease and the longitudinal design of the study, we chose to use the proxy of the patient to rate his or her HRQoL. It is known that factors affecting the relationship between the proxy and the patient may influence the proxy’s perception of the HRQoL of the patient [23]. In addition, cognitive functioning is primarily affected by dementia and proxies may, therefore, be less capable of providing valid HRQoL ratings. However, this is true for both groups, and cannot explain the variations in HRQoL. What might explain the variance is that different self-report measures were used in both groups. In the reference group, face-to-face interviews were used; in the GNP group, postal questionnaires were used. The method of collecting self-report data may have influenced the representativeness and quality of the data. Hanmer and colleagues found that interviewer-administered surveys seem to have higher reports of HRQoL than self-administered surveys [24]. In addition, in the case of face-to-face interviews, the presence of an interviewer can cause bias, as this might be distracting for the respondent [25].

A weakness of this study is that data from the reference sample were collected at an earlier time phase. There is an 8-year time lag between the data collection for the reference sample and the GNP group. Although the organization, policy and procedures within the DOC-PG have not changed during this period, new developments in the care for patients with dementia more generally may have been implemented during this time. In addition, changes in GP attitudes and education might have affected the results of the patient and informal caregiver level. Other limitations are that outcome data on patient and informal caregiver level in the GNP group were collected by means of questionnaires, which were sent to the informal caregiver. In the reference sample, data were collected by means of face-to-face interviews. This might explain the higher dropout rates in the GNP group. In addition, patient data were rated by their proxy and may have been biased (e.g., through care burden), but this would apply to both groups. With respect to GP concordance, the period between the DOC-PG assessment and concordance check was relatively long and varied from 3 to 20 months. Again, this applies to both groups. Partly, as a result of the methodological weakness of the study, we cannot claim for sure that the improved adherence to advice reduced informal caregiver burden. Additional (e.g., randomized) research should further confirm whether adding a GNP could be beneficial for patients with dementia and their informal caregivers in terms of reduced burden and improved quality of life.

## Conclusion

This study suggests that adding a GNP to an outpatient diagnostic multidisciplinary facility for patients with cognitive disorders (DOC-PG) may improve the quality of care in terms of an improved adherence to advice given by the DOC-PG team.

## Abbreviations

GP: general practitioner
GNP: geriatric nurse practitioner
DOC-PG: Diagnostic Observation Center for PsychoGeriatry
HRQoL: health-related quality of life
CarerQoL: care-related quality of life
VAS: visual analogue scale
MMSE: mini-mental state examination
